# Clinically Defined Mutations in *MEN1* Alter Its Tumor-suppressive Function Through Increased Menin Turnover

**DOI:** 10.1158/2767-9764.CRC-22-0522

**Published:** 2023-07-24

**Authors:** Suzann Duan, Sulaiman Sheriff, Uloma B. Elvis-Offiah, Brandon L. Witten, Travis W. Sawyer, Sinju Sundaresan, Tomasz Cierpicki, Jolanta Grembecka, Juanita L. Merchant

**Affiliations:** 1 Division of Gastroenterology and Hepatology, Department of Medicine, University of Arizona College of Medicine, Tucson, Arizona.; 2 Department of Physiology, University of Arizona College of Medicine, Tucson, Arizona.; 3 Department of Optical Sciences, University of Arizona Wyant College of Optical Sciences, Tucson, Arizona.; 4 Department of Physiology, Midwestern University, Downers Grove, Illinois.; 5 Department of Pathology, University of Michigan, Ann Arbor, Michigan.

## Abstract

Loss of the tumor suppressor protein menin is a critical event underlying the formation of neuroendocrine tumors (NET) in hormone-expressing tissues including gastrinomas. While aberrant expression of menin impairs its tumor suppression, few studies explore the structure–function relationship of clinical multiple endocrine neoplasia, type 1 (*MEN1*) mutations in the absence of a complete LOH at both loci. Here, we determined whether clinical *MEN1* mutations render nuclear menin unstable and lead to its functional inactivation. We studied the structural and functional implications of two clinical *MEN1* mutations (R516fs, E235K) and a third variant (A541T) recently identified in 10 patients with gastroenteropancreatic (GEP)-NETs. We evaluated the subcellular localization and half-lives of the mutants and variant in *Men1*-null mouse embryo fibroblast cells and in hormone-expressing human gastric adenocarcinoma and NET cell lines. Loss of menin function was assessed by cell proliferation and gastrin gene expression assays. Finally, we evaluated the effect of the small-molecule compound MI-503 on stabilizing nuclear menin expression and function *in vitro* and in a previously reported mouse model of gastric NET development. Both the R516fs and E235K mutants exhibited severe defects in total and subcellular expression of menin, and this was consistent with reduced half-lives of these mutants. Mutated menin proteins exhibited loss of function in suppressing tumor cell proliferation and gastrin expression. Treatment with MI-503 rescued nuclear menin expression and attenuated hypergastrinemia and gastric hyperplasia in NET-bearing mice. Clinically defined *MEN1* mutations and a germline variant confer pathogenicity by destabilizing nuclear menin expression.

**Significance:**

We examined the function of somatic and germline mutations and a variant of *MEN1* sequenced from gastroenteropancreatic NETs. We report that these mutations and variant promote tumor cell growth and gastrin expression by rendering menin protein unstable and prone to increased degradation. We demonstrate that the menin-MLL (mixed lineage leukemia) inhibitor MI-503 restores menin protein expression and function *in vitro* and *in vivo*, suggesting a potential novel therapeutic approach to target MEN1 GEP-NETs.

## Introduction

Between 20% and 40% of gastroenteropancreatic neuroendocrine tumors (GEP-NET) are associated with inactivating mutations in the multiple endocrine neoplasia I (*MEN1*) gene encoding the tumor suppressor protein menin (1–3). Both sporadic and familial forms of the hereditary MEN1 syndrome mark a strongly penetrant and autosomal dominant disorder characterized by the development of tumors in the parathyroid glands, anterior pituitary, endocrine pancreas, and gastrointestinal tract (4–6). GEP-NETs that secrete the gastric peptide hormone gastrin (i.e.*,* gastrinoma) comprise the most clinically aggressive of *MEN1* cases, with 60% of patients presenting with lymph node metastases upon diagnosis (7). To date, approximately 1,700 unique mutations have been identified across the coding region of the *MEN1* (11q13) locus; however, mutational hotspots and a clear genotype–phenotype correlation remain ambiguous (8). Frameshift insertions and deletions comprise the majority of *MEN1* mutations (40%), while missense mutations, nonsense mutations, and splice site defects account for 25%, 10%, and 11% of the remaining mutations, respectively (9).

Originally modeled by Knudsons’ “two hit” hypothesis, complete *MEN1* inactivation leading to tumor development is thought to require an additive somatic *MEN1* mutation in the context of an existing (i.e., inherited) germline defect (10). However, a study of MEN1-gastrinomas showed that LOH at the 11q13 locus occurred in only 46% of cases, with either partial, complete, or no LOH observed in small synchronous tumors from the same patient (11). Moreover, hyperplastic precancerous lesions in MEN1 patients with multifocal gastrin and somatostatin-secreting NETs retain both *MEN1* alleles, suggesting that an independent hit leading to allelic inactivation is a critical step in the pathogenesis of these tumors (11). These studies raise the possibility that alternative posttranslational mechanisms regulate menin protein expression and function in the absence of complete LOH. Indeed, we and others have shown that menin is tightly regulated by extracellular cues including gastrin and serum-derived growth factors (12, 13).

Menin negatively regulates the expression of genes involved in cell proliferation and endocrine cell specification (e.g., gastrin) by acting as a nuclear adaptor protein and recruiting transcriptional regulators to promoter binding sites (e.g., JUND and mixed lineage leukemia I/MLL; refs. 14–17). Thus, prior research has centered on mapping clinical *MEN1* mutations to specific functional domains and regulatory sequences that might preclude menin from interacting with known nuclear-binding partners. Targeted deletion studies have identified two nuclear export signals (NES1 and NES2) and three nuclear localization signal sequences (NLS1, NLSa, and NLS2) that are critical for maintaining proper localization of menin to the nucleus (18, 19). While aberrant subcellular expression of menin impairs its tumor suppressor function, few studies have explored the structure–function relationship of clinical *MEN1* mutations within this context.

We recently performed whole-exome sequencing (WES) on a cohort of 10 patients with confirmed GEP-NETs and identified a *MEN1* germline variant or germline and somatic mutations in all subjects (20). We mapped these to coding regions within the *MEN1* locus and focused on two mutations and a third variant occurring in the central pocket and NLS regions with the potential to destabilize menin protein. Here we investigated the hypothesis that clinically defined *MEN1* mutations or variants confer pathogenicity by destabilizing nuclear menin.

## Materials and Methods

### Plasmid Design and Purification

Plasmid design and construction was based on the original human *MEN1* sequence first reported by Chandrasekharappa and colleagues and annotated in the GenBank database (U93236; ref. 21). This coding region of the human *MEN1* gene (1,830 nucleotides) was ligated to the EcoRI site of the pcDNA3.1+/C-(K)-DYK plasmid upstream of the FLAG-epitope DYKDDDDK sequence. Two single-nucleotide mutations and a third single-nucleotide variant previously identified by WES of patients with GEP-NETs (20) were introduced as follows: c.1546dupC (R516fs), c.703G>A (E235K), and c.1621G>A (A541T). As the c.1546dupC mutation encodes a premature stop codon that results in protein truncation at amino acid 529, the FLAG-epitope sequence was introduced at the N-terminal domain. Plasmid construction and validation were performed by GenScript (Genscript Biotech Corp). BL21 (DE3) competent cells (Invitrogen) were transformed with the pcDNA empty vector or the vector expressing wild-type *MEN1* containing the respective mutations. Plasmids were purified from transformed *Escherichia coli* following ampicillin selection using the QIAGEN Plasmid Purification Midi-Prep Kit (QIAGEN) and reconstituted in sterile Tris-EDTA buffer.

### Cell Culture

*Men1-*null mouse embryo fibroblasts (*MEF^ΔMen1^*) were a gift from Dr. Agarwal (NIH, Bethesda, MD). *MEF^ΔMen1^* cells and wild-type MEFs were grown in DMEM containing 10% FBS (Sigma Millipore) and penicillin-streptomycin antibiotic. The BON-1 human pancreatic NET (PNET) cell line and AGS, MKN-45G, and KATO III human gastric adenocarcinoma cell lines were purchased from ATCC. BON-1 and AGS cells were grown in DMEM/Ham's F12 media supplemented with 5% FBS, 5% newborn calf serum, and penicillin-streptomycin. MKN-45G cells were grown in RPMI1640 media containing l-glutamine and supplemented with 5% FBS and penicillin-streptomycin. KATO III cells were grown in Iscove's modified Dulbecco's medium media supplemented with 10% FBS and penicillin-streptomycin. The previously reported on GLUTag murine enteroendocrine tumor cell line (22, 23) was grown in DMEM supplemented with 10% FBS and penicillin-streptomycin. All cells were maintained at 37°C and used at passage numbers 3–10. Cells were routinely tested every 6 months for *Mycoplasma* contamination using the Universal *Mycoplasma* Testing kit (catalog no. 30-1012K, ATCC) prior to initiating experiments.

### Subcellular Fractionation

Within 24 hours of seeding cells into 6-well plates, cells were transfected with 1.2 μg of the FLAG vectors using Polyplus jetOPTIMUS reagent (Polyplus). Following 48 hours of transfection, AGS, MKN-45G, and GLUTag cell lines were washed twice in PBS and mechanically dissociated. Cells were centrifuged for 5 minutes at 500 × *g* at 4℃ and then the pellet was resuspended in a hypotonic lysis buffer consisting of 20 mmol/L HEPES, 0.1% IGEPAL, 1 mmol/L dithiothriotol (DTT), and 1X HALT protease/phosphatase inhibitor (Thermo Fisher Scientific). The pellet was pipetted up and down 15–20X and incubated on ice for 10 minutes prior to pulse vortexing for 10 seconds. The cells were centrifuged for 5 minutes at 15,000 × *g* at 4℃ and the suspension was collected as the cytoplasmic protein fraction. The pellet was resuspended in a hypertonic lysis buffer consisting of 20 mmol/L HEPES, 20% glycerol, 500 mmol/L NaCl, 1.5 mmol/L MgCl_2_, 0.2 mmol/L EDTA, 0.1% IGEPAL, 1 mmol/L DTT, and protease/phosphatase inhibitor. The protein solution was vortexed, then incubated on ice for 1 hour with intervals of vortexing every 15 minutes. The solution was centrifuged at 15,000 × *g* for 15 minutes at 4℃ and the supernatant was collected as the nuclear protein fraction. Volumes for lysis buffers were scaled at a 4:1 ratio to account for the total protein concentration in each subcellular compartment.

For MEF*^ΔMen1^* cells, subcellular protein extracts were obtained by using the NE-PER Nuclear and Cytoplasmic Extraction Kit (catalog no. 78833, Thermo Fisher Scientific). Transfected cells were washed with PBS and trypsinized for 1 minute using Trypsin-EDTA solution (Invitrogen). The cells were centrifuged for 5 minutes at 500 × *g* and pellets were washed with PBS prior to subcellular fractionation. Pellets were resuspended in cytoplasmic extraction reagent I buffer (0.1 mL) supplemented with protease/phosphatase inhibitor and the cytoplasmic protein fraction was obtained by following kit instructions. Nuclear extraction reagent buffer (0.05 mL) was added to remaining nuclear pellets and proteins were extracted according to manual instructions.

### Western Blot Analysis

Protein extracts (10–15 μg) were run on a precast gradient gel (4%–12% Bis-Tris, Invitrogen) for either 75 minutes at 100 V or 65 minutes at 140 V. Proteins were transferred onto a polyvinylidene difluoride membrane using the iBlot-2 transfer system (Invitrogen). The membrane was washed and blocked with 5% BSA in TBS with 0.05% Tween-20 (TBST) buffer for 1 hour at 24℃. Blots were then probed overnight at 4℃ with the following antibodies diluted in 5% BSA-TBST: Mouse anti-FLAG mAb (1:10,000 dilution, catalog no. F1804, Clone M2, RRID: AB_262044, Sigma Millipore), Mouse anti-Menin monoclonal antibody (1:2,000 dilution, catalog no. sc-374371, Clone B-9, RRID: AB_10987907, Santa Cruz Biotechnology), Rabbit anti-Menin polyclonal (1:10,000 dilution, catalog no. A300-105A, RRID: AB_2143306, Bethyl Laboratories). Membranes were washed three times in TBST, then incubated in horseradish peroxidase (HRP)-linked anti-mouse or anti-rabbit IgG antibody for 1 hour at 24℃ with gentle rocking (1:5,000 dilution, Cell Signaling Technology). Membranes were washed in TBST and protein bands were visualized using the Pierce ECL detection system (catalog no. 32106, Thermo Fisher Scientific). For loading controls, membranes were re-probed with the following antibodies diluted in 5% BSA TBST for 1 hour at 24℃: Rabbit anti-GAPDH mAb (1:5,000 dilution, catalog no. 5174, Clone D16H11, RRID: AB_10828810, Cell Signaling Technology), Rabbit anti-β-Tubulin HRP-conjugated antibody (1:2,000 dilution, catalog no. 5346, Clone 9F3, RRID: AB_1950376, Cell Signaling Technology), Rabbit anti-Histone H3 HRP-conjugated antibody (1:10,000 dilution, catalog no. 12648, Clone D1H2, RRID: AB_2797978, Cell Signaling Technology).

For quantitation, films were scanned, converted to gray scale, and quantified using FIJI software (ref. 24; open source). Protein bands were identified by their expected molecular weights and quantified by equal area selection using the inverted mean gray value (MGV) method. MGVs were subtracted from 255 (pixel density maximum) to obtain the inverted MGV, then background subtraction and normalization to respective loading controls was applied. Representative images of blots were enhanced using global brightness adjustments that were applied to the entire image.

### Immunocytochemistry and Immunofluorescence Staining

AGS, MKN-45G, GLUTag, and MEF*^ΔMen1^* cells were seeded onto glass coverslips and incubated overnight to allow the cells to adhere to coverslips. Cells were transfected with FLAG-epitope expressing plasmid using Polyplus jetOPTIMUS transfection reagent as described previously. Following 48 hours, cells were washed twice with PBS and fixed in 4% paraformaldehyde (PFA) for 15 minutes at 24℃. Cells were washed prior to permeabilization with 0.5% Triton-X in TBST for 10 minutes at 24℃. Coverslips were blocked for 1 hour at 24℃ with 10% donkey serum in 0.1% BSA, 0.1% Triton-X TBST buffer, then incubated in Rabbit anti-FLAG primary antibody (1:400 dilution, catalog no. 14793, Clone D6W5B, RRID: AB_2572291, Cell Signaling Technology) overnight at 4℃ in a humid chamber. Coverslips were washed in TBST and then incubated in Donkey anti-Rabbit IgG Alexa Fluor-conjugated 594 secondary antibody (1:500 dilution, catalog no. A21207, RRID: AB_141637, Invitrogen) for 1 hour at 24℃ in a humid chamber. Coverslips were washed in TBS, mounted with Prolong Gold anti-fade mounting medium with 4′, 6-diamidino-2-phenylindole (DAPI; Invitrogen), and imaged using the Nikon Olympus epifluorescence microscope with Cellsense software. For quantitation of nuclear and cytoplasmic expression, 10 images at 200X magnification were acquired per plasmid condition per experimental replicate. A total of 30 images per plasmid per condition were quantified on the basis of distinct morphologic localization of menin to the nucleus and analyzed for statistical significance using Graphpad Prism 9 software. Margins were determined by analyzing images of wild-type menin expressed in each cell type showing a crisp rounded morphology colocalized with DAPI.

### Half-life Study

MEF*^ΔMen1^* cells were seeded onto a 6-well plate at a density of 0.5 × 10^6^ cells per well. Cells were transfected 24 hours later with 1.2 μg of respective FLAG epitope-expressing plasmids using the Polyplus jetOPTIMUS reagent as described previously (Polyplus). After 40 hours, fresh media with cycloheximide was added (10 μmol/L, Tocris). In parallel, cells were pretreated with the proteosome inhibitor MG-132 (10 μmol/L, Cell Signaling Technology) for 1 hour prior to the addition of cycloheximide. Treated cells were harvested at timepoints ranging from 0 to 8 hours. Cells were washed with ice-cold PBS and lysed in 0.1 mL of RIPA buffer supplemented with protease and phosphatase inhibitors (Thermo Fisher Scientific). Cell lysates were passed through a 27 G syringe needle five times, incubated on ice for 15 minutes, and the resulting cell homogenate was centrifuged at 10,000 × *g* for 15 minutes at 4°C. Supernatant protein samples were used for determination of the half-life by Western blot analysis as described previously.

Inverted MGVs for protein bands were quantified as described in the previous section and expression values were normalized to respective GAPDH bands following background subtraction. The half-life was calculated by plotting normalized expression values as a function of time and fitting an exponential decay function to expression values above 0 (*y* = e^−^*^kx^*). The decay constant slope (*k*) from the fit was used to calculate the half-life by finding the time required for the signal level to drop to 0.5, expressed by the equation (*x* = −ln(0.5)/*k*), where *x* is the half-life (24).

### Cell Proliferation

For crystal violet staining, AGS, MKN-45, and GLUTag cells were seeded at a density of 25,000 cells in 24-well plates. Cells were transfected with 0.3 μg of respective plasmid 24 hours postseeding using Polyplus jetOPTIMUS reagent as described previously. Growth media was changed 24 hours following transfection and proliferation was assessed at 72 hours posttransfection by crystal violet staining. Cells were washed with PBS prior to fixing in 4% PFA for 15 minutes at 24℃. Cells were washed in PBS and incubated in 0.1% crystal violet dye for 20 minutes at 24℃. Cells were washed with distilled water and allowed to dry prior to imaging on the ECHO Revolve inverted microscope (ECHO).

Quantitative assessment of cell proliferation was accomplished by evaluating bromodeoxyuridine (BrdU) incorporation as described by the manufacturer (Cell Signaling Technology, #6813). Cells were seeded at a density of 10,000 cells in 96-well plates and transfected after 24 hours with respective menin plasmids as described previously. 1X BrdU was added to each well and allowed to incorporate into cells overnight at 24, 48, and 120 hours posttransfection. Cells were fixed and denatured for 30 minutes, incubated with anti-BrdU antibody (included in the kit) for 1 hour at 24°C, washed, and incubated in HRP-conjugated secondary antibody for 30 minutes at 24°C. Samples were washed, incubated in 3,3′,5,5′ tetramethylbenzidine (TMB) substrate, and the HRP reaction was stopped and visualized by measuring the optical density (OD) at 450 nm using the Gen5 Microplate Reader and Data Analysis Software (BioTec). OD values were normalized to the empty pcDNA vector at the respective matching timepoint to adjust for technical variations and cell seeding density between experimental replicates.

### Gastrin-Luciferase Promoter Assay

The gastrin-luciferase (GasLuc) plasmid reporter system was used to evaluate transcriptional activity at the gastrin promoter, as reported previously. A 240 bp sequence of the human gastrin gene (25) was cloned upstream of the firefly luciferase-encoding sequence in the pGL3B reporter plasmid (catalog no. E1751, Promega). Upon reaching 60%–75% confluency in 6-well plates, AGS cells were cotransfected with 0.75 μg of the GasLuc plasmid construct and respective FLAG-epitope expressing menin plasmids using jetOPTIMUS transfection reagent as described previously. To account for transfection efficiency, the PRL-TK *Renilla* Luciferase Reporter Vector (Promega) was also cotransfected into cells. After 24 hours posttransfection, cells were serum starved for 24 hours, then treated with recombinant human EGF (40 nmol/L) for 16 hours. The EGF concentration was selected on the basis of prior studies evaluating EGF-mediated induction of gastrin gene expression (26). Cells were then lysed and luciferase activity was determined using the Dual-Luciferase Assay System (catalog no. E1980, Promega). Luminescence was measured using the Synergy 2 Microplate Reader with Gen5 analysis software (BioTec).

### Quantitative PCR

Cells were harvested at 48 or 72 hours posttransfection to evaluate *MEN1* and *GAST* mRNA. For the EGF studies, cells were transfected for 24 hours, serum starved for 24 hours in 2% FBS, and then treated with 40 nmol/L of recombinant human EGF for 16 hours prior to lysis and RNA extraction. Total mRNA was extracted from cells using the Purelink RNA Isolation kit (Invitrogen). Cells were lysed in RNA lysis buffer containing 1% beta-mercaptoethanol and homogenized by passing the lysate through a 20G syringe seven times. The lysate was mixed with equal volumes of 70% ethanol prior to adding to the Purelink spin columns for RNA extraction. Up to 1 μg of RNA was used for cDNA synthesis and qRT-PCR. RNA was incubated with ezDNase enzyme mix for 2 minutes at 37℃ to digest contaminating genomic DNA prior to performing cDNA synthesis using Superscript VILO Master Mix (Thermo Fisher Scientific). qRT-PCR was performed using PowerUp Syber Green Master Mix with 5 ng of cDNA added per reaction (Invitrogen). qRT-PCR thermocycling conditions are as follows: 95℃ for 2 minutes followed by 40 cycles of 95℃ for 15 seconds and 60℃ for 1 minutes (Bio-Rad). The following Prime Time ready-made primers from Integrated DNA Technologies were used at 500 nmol/L:

*GAST:* GCAGCGACTATGTGTGTATGT (forward)
*GAST:* CCATCCATAGGCTTCTTCTTCTT (reverse)
*MEN1:* GTGCCTAGTGTGGGATGTAAG (forward)
*MEN1:* TGAAGAAGTGGGTCATGGATAAG (reverse)
*HPRT1:* TCCCAAAGTGCTGGGATTAC (forward)
*HPRT1:* CCCAGTCCATTACCCTGTTTAG (reverse)

### Animals

All animal experiments were approved by the University of Michigan's Committee on the Use and Care of Animals. *VC:Men1^FL/FL^; Sst^−/−^*(*Men1^ΔIEC^; Sst^−/−^*) mice were generated as previously described by breeding the B6.Cg-Tg(Vil1-cre)1000Gum/(Strain #021504, RRID:IMSR_JAX:021504) mouse strain to the 129S(FVB)-Men1tm1.2Ctre/J (Strain #005109, RRID:IMSR_JAX:005109) strain (12). Mice were housed in a facility with access to food and water *ad libitum*. Experimental mice were fed omeprazole-laced chow (200 ppm, TestDiet) for 6 months to 1 year. Vehicle (25% DMSO, 25% PEG400, 50% PBS) or MI-503 (10, 30, and 55 mg/kg body weight) was administrated intraperitoneally, once daily for a total of 4 weeks. Blood was drawn via submandibular bleeding prior to MI-503 administration to determine basal plasma gastrin levels and at the end of MI-503 treatment. Mouse genders used were equivalently distributed across the control and treatment groups. Approximately 8 mice were included in each treatment group for the *in vivo* studies.

### Gastrin Plasma Measurement

Mice were fasted for 16 hours prior to euthanasia. Blood was collected by cardiac puncture in heparin-coated tubes and plasma was collected by centrifugation at 5,000 × *g* for 10 minutes at 4°C. Approximately 50 μL of the plasma was used for measuring gastrin levels using the Human/Mouse/Rat Gastrin-I Enzyme Immunoassay Kit (catalog no. EIAM-GAS-1, RayBiotech). For measuring gastrin content of primary glial cultures, cultures were scraped in PBS using cell scraper. Cells were pelleted, and then boiled in deionized water (volume equals to 10 times the size of the pellet). The extract was spun briefly as described above and supernatant was used for gastrin measurements. Gastrin content was expressed after normalization to total protein content.

### IHC (Fixed Tissues)

Duodenums of mice were flushed with ice-cold PBS, cut open and fixed overnight in 10% formaldehyde solution at 4°C. They were then transferred to 70% ethanol and processed before embedding in paraffin (TissueTek). Five μm sections were cut from paraffin blocks for staining. Sections were deparaffinized in xylene and dehydrated by brief sequential incubations in 70%, 90%, and 100% ethanol. Heat-mediated antigen retrieval was performed by heating the slides to 95°C in Tris-EDTA (pH 9.0, catalog no. ab93684, Abcam), or sodium citrate (pH 6.0, catalog no. ab93678, Abcam) for 30 minutes. Nonspecific binding was blocked by incubation in 10% donkey serum followed by incubation with primary antibodies overnight at 4°C. Goat anti-gastrin (catalog no. sc-7783; RRID:AB_2108261, Santa Cruz Biotechnology) was diluted to 1:1,000 and rabbit anti-menin (catalog no. A300-105A, RRID:AB_2143306, Bethyl Laboratories) was diluted 1:10,000 in blocking buffer. For menin staining, tyramide signal amplification (TSA) was used (catalog no. NEL76300, PerkinElmer) according to manufacturer instructions. For fluorescent detection, sections were incubated with Alexa Fluor-conjugated secondary antibodies for 30 minutes (1:400 dilution, Invitrogen) at 24°C. ProLong Gold antifade reagent with DAPI (Invitrogen) was used for nuclear counterstaining. Images were taken using a Nikon inverted confocal microscope using Olympus CellSens Imaging software (Nikon).

Primary glial cultures were plated on laminin and poly-D-lysine–coated coverslips, fixed using 4% paraformaldehyde for 20 minutes at 24°C, and then permeabilized for 5 minutes with 0.2% Triton X-100/PBS. Cultures were then blocked in 1% serum (from the appropriate species) for 30 minutes at 24°C, incubated in primary antibodies overnight at 4°C, and stained as described above. Primary antibodies were prepared in blocking reagent and include: goat anti-gastrin (catalog no. sc-7783; RRID:AB_2108261, Santa Cruz Biotechnology) used at 1:1,000 dilution and rabbit anti-menin primary antibody (catalog no. A300-105A, RRID:AB_2143306, Bethyl Laboratories) used at 1:10,000 dilution with the TSA staining kit (catalog no. NEL701A001KT, PerkinElmer) according to manufacturer instructions.

### Primary Glial Culture Isolation

The first 6–8 cm of the proximal duodenum from 2 mice were used for the isolation of duodenal enteric glia and pooled as described previously (27). Briefly, the longitudinal muscle/myenteric plexus attached to the submucosa was removed from the underlying circular muscle by gently rubbing the edges of forceps and teasing it away using a cotton swab wetted with Dulbecco's Phosphate Buffered Saline (DPBS) without calcium and magnesium by applying light horizontal strokes. Next, the epithelium was removed by incubating the tissue twice in EDTA/HEPES/DPBS (5 mmol/L/10 mmol/L) solution at 4°C for 10 minutes with gentle rocking. The tissue was gently triturated five times using a 5 mL pipette to dislodge the epithelium. The tissue was recovered on a 100 μm filter. After the last incubation, epithelial-stripped tissue was incubated in Cell Recovery Solution (Corning) for 30 minutes at 4°C with rocking then triturated three times to generate the glial cell containing solution, filtered through a 40 μm filter to retain the tissue and large clumps. The cell suspension that flows through was spun at 2,000 × *g* for 5 minutes at 4°C and then resuspended in 1.2 mL of DMEM/F12 medium supplemented with 10% FBS, 100 IU/mL penicillin, 100 μg/mL streptomycin, 20 μg/mL gentamicin, 6 mmol/L glutamine, and 2.1 g/L NaHCO_3_. About 100–200 μL of dissociated cell suspension was transferred to laminin and poly-D-lysine–coated 6-well plate containing DMEM/F12 medium supplemented with B-27, 2 mmol/L l-glutamine, 1% FBS, 10 μg/mL glial cell-derived neurotrophic factor, 100 IU/mL penicillin, 100 μg/mL streptomycin, and 20 μg/mL gentamicin. The cells attach by 16 hours. After changing the media within 24 hours of attachment, the cells remain viable for 4 days.

### Statistical Analysis

Unpaired Student *t* test was used to test for significance between two groups. One-way ANOVA with Tukey *post hoc* test was used for comparisons of three or more groups. Two-way ANOVA with Tukey *post hoc* test was used for analysis of two or more groups when comparing more than one condition. For timed assays, pairwise comparisons across plasmid conditions were restricted to each respective timepoint. All quantitative PCR data, luciferase data, and BrdU studies reflect three or more experimental replicates, in which data were normalized to the negative control group for each experiment to account for technical variation.

### Data Availability Statement

The data generated in this study are available upon reasonable request from the corresponding author.

## Results

### WES of Patient-derived GEP-NETs Identifies Germline and Somatic Mutations and Variants in the Human *MEN1* Gene

**FIGURE 1:**
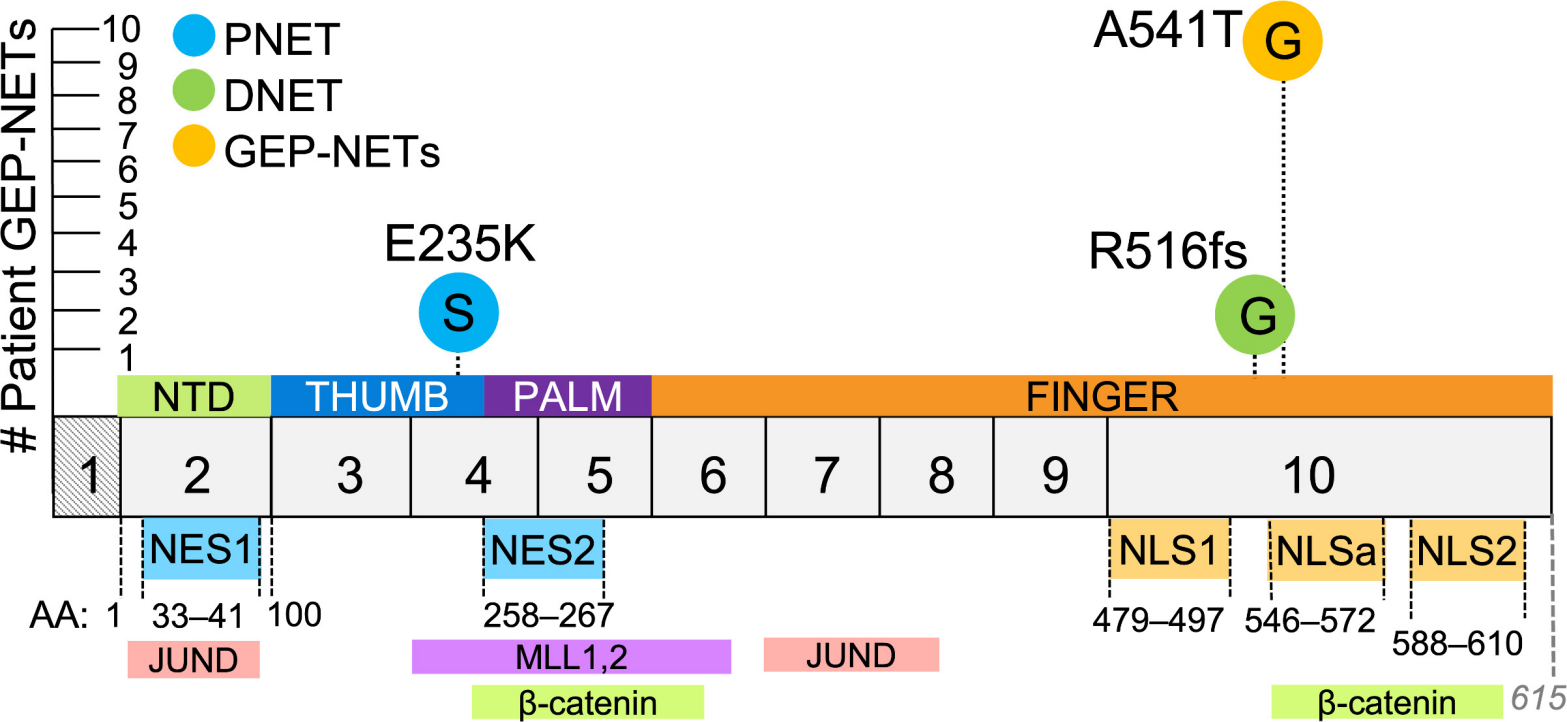
WES of patient-derived GEP-NETs identifies germline and somatic mutations in the human *MEN1* gene. Germline and somatic *MEN1* mutations and variants were previously identified by WES of matched buffy coat and tumor specimens from 9 patients with clinically confirmed GEP-NETs (20). A 10th patient was included in the study analysis following clinical testing for *MEN1*. We focused our investigation on three single-nucleotide mutations: a c.1546dupC germline mutation identified in a duodenal NET (DNET) that results in a frameshift mutation and introduction of an arginine at amino acid (aa) 516 (R516fs); a c.703G>A somatic mutation identified in a PNET resulting in a glutamic acid to lysine substitution at aa 235 (E235K); and a c.1621G>A germline polymorphism identified in all nine patient samples that underwent WES. In this patient cohort, the c.1621G>A polymorphism results in an alanine to threonine substitution at aa 541 (A541T) immediately upstream to an NLSa. Menin has three NLS sequences in exon 10 and two nuclear export signal (NES) sequences. G = germline, S = somatic, aa = amino acid, NES = nuclear export signal, NLS = nuclear localization signal, NTD = N-terminal domain. Exon1 (hatched) is transcribed but not translated.

WES was previously performed on matched buffy coat and tumor specimens from 9 patients with clinically confirmed GEP-NETs (20). A 10th patient was included in the study analysis following clinical testing for the MEN1 syndrome. GEP-NETs consisted of five duodenal NETs, of which four cases were confirmed gastrinomas (DGAST), and five PNETs with one confirmed gastrinoma (PGAST). From these analyses, we identified and mapped a germline variant in 9 patients and novel somatic and germline mutations in tumor specimens collected from 2 patients (Fig. 1). The variant and two mutations were all identified as heterozygous in tumors from this patient cohort. A previously reported single-nucleotide variant (polymorphism) at c.1621G>A (28–30) was detected in all nine patient samples that underwent WES and occurred irrespective of tumor location. This variant was identified using the original human *MEN1* sequence first reported by Chandrasekharappa and colleagues and is annotated in the GenBank database (U93236; ref. 21). The resulting GCA>ACA codon changes the wild-type amino acid at residue 541 from an alanine to a threonine (A541T) and lies immediately upstream of an accessory NLS sequence (NLSa, aa 546–572), one of the three NLS sequences located in exon 10 (Fig. 1). We further identified frameshift mutations in two DGASTs and two PNETs spanning exons 2, 3, 8, and 10 which translated into a truncated protein. Of these, we focused on a germline DGAST-specific single-nucleotide insertion identified in exon 10 (c.1546dupC) that results in a premature stop codon at residue 516 (R516fs). In addition to the C-terminal mutation and variant, we included in our analyses a PNET-specific somatic missense mutation (c.703G>A) that was mapped to exon 4. This single-nucleotide mutation substitutes a negatively charged glutamic acid for a positively charged lysine residue (E235K) and occurs upstream of a nuclear export signal sequence (NES2, aa 258–267; Fig. 1). We next investigated whether these two mutations and variant confer altered structural and functional properties that preclude menin from functioning as a negative regulator of NET development.

### Tumor-acquired Mutations in *MEN1* Result in Aberrant Subcellular Localization at the Protein Level

We reasoned that clinical *MEN1* mutations occurring near the NLS and NES sequences may affect the subcellular localization of menin. As a nuclear scaffold protein, proper localization of menin to the nucleus is critical for regulating the expression of genes involved in cell proliferation and endocrine cell specification (18, 25, 31). To evaluate whether the R516fs and E235K mutations and A541T variant altered subcellular menin expression, we constructed plasmids harboring the two mutations and variant within the 1,830 bp of the coding human *MEN1* sequence originally reported by Chandrasekharappa and colleagues (21). The FLAG-epitope was located at the C-terminus for the point mutation and single-nucleotide variant to distinguish exogenously expressed menin from the endogenous protein. Because the R516fs mutation results in premature protein truncation at 529 aa, we inserted the FLAG-epitope in the N-terminal domain of the R516fs construct.

**FIGURE 2:**
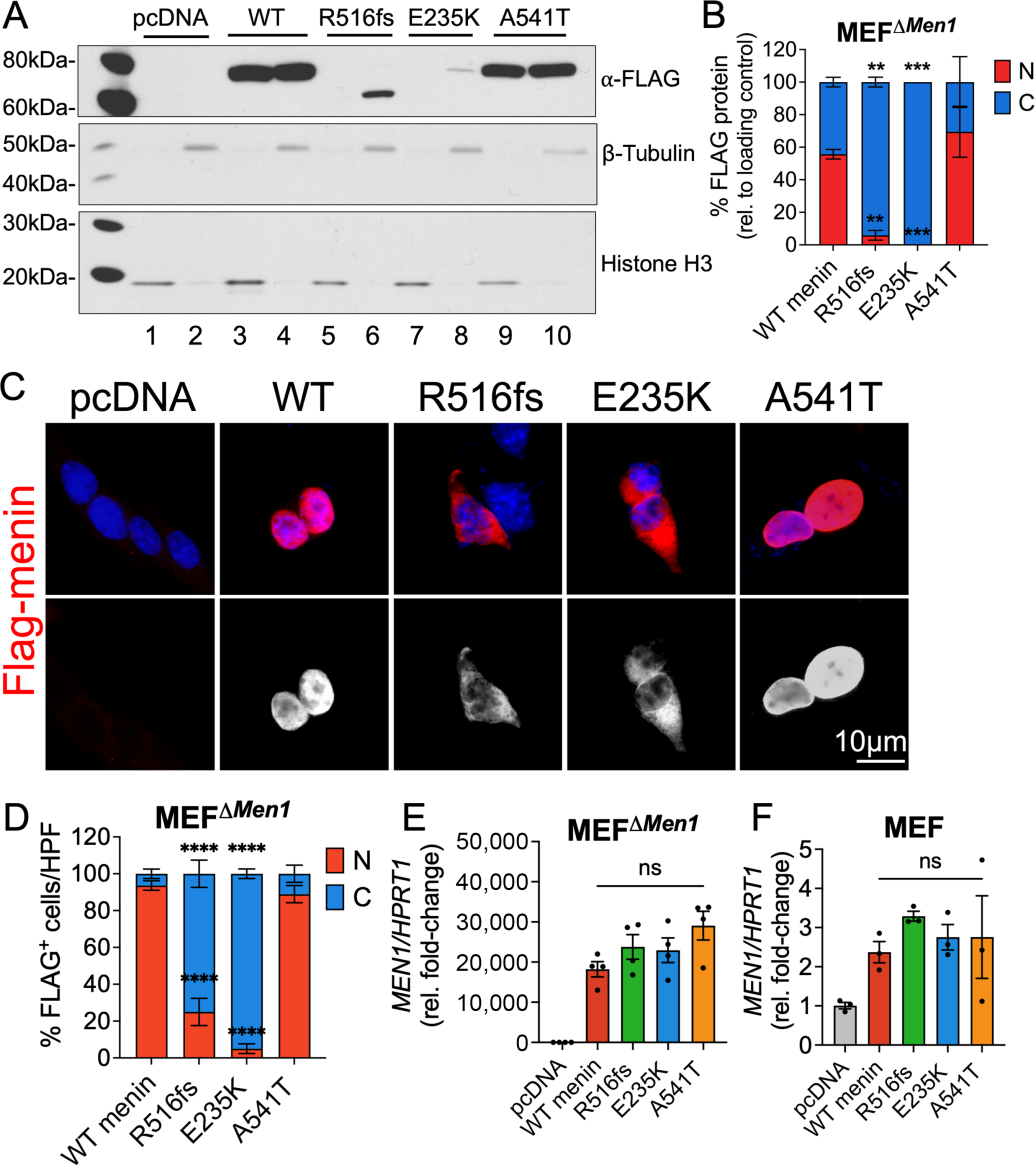
Tumor-acquired mutations in *MEN1* result in aberrant subcellular localization of menin at the protein level. Empty vector (pcDNA), wild-type menin, and menin mutant proteins bearing FLAG-epitope tags were transiently overexpressed in *Men1*-null mouse embryonic fibroblasts (MEF*^ΔMen1^*). **A,** Western blot analysis of nuclear and cytoplasmic protein extracts probed for FLAG expression. Nuclear fractions are shown in lanes 1, 3, 5, 7, and 9 while cytoplasmic fractions are shown in lanes 2, 4, 6, 8, and 10. Histone H3 and β-tubulin were used as loading control markers for nuclear and cytoplasmic fractions, respectively. **B,** Quantitation of FLAG protein band expression in nuclear and cytoplasmic compartments normalized to respective loading controls. *n* = 3 experimental replicates; **, *P* < 0.01; ***, *P* < 0.001 by two-way ANOVA with Tukey *post hoc* test; mean ± SEM. **C,** Immunofluorescent images of FLAG-stained MEF*^ΔMen1^*cells (red) costained with DAPI to visualize the nucleus (blue). Bottom panel shows FLAG staining in white. Scale bar = 10 μm. **D,** Quantitation of FLAG expression in nuclear and cytoplasmic compartments expressed as a percentage of positively transfected cells. Ten images taken at 200X magnification were counted across *n* = 3 experiments for a total of 30 images per group; *, *P* < 0.05 by two-way ANOVA with Tukey *post hoc* test; mean ± SEM. **E,** Relative *Men1* mRNA expression in menin-null MEFs following overexpression of empty vector, wild-type menin, and the three mutants. **F,** Relative *Men1* mRNA expression in wild-type MEFs expressing endogenous menin as a control. *n* = 3–4 experimental replicates; n.s. by one-way ANOVA. WT, wild-type.

Characterization was performed in *Men1*-null mouse embryonic fibroblasts (MEF*^ΔMen1^*) to study potential structural deficits conferred by the respective mutations in the absence of any known interaction with endogenously expressed menin (32). Wild-type menin and the three mutated menin proteins were transiently overexpressed in MEF*^ΔMen1^* cells and protein expression was evaluated by subcellular fractionation and Western blot analysis. As reported previously, wild-type menin localized to both nuclear, perinuclear, and cytoplasmic compartments, with near-equal distribution observed by biochemical fractionation (Fig. 2A and B). A similar expression pattern was observed in cells expressing the A541T variant. In contrast, the expression of both R516fs and E235K mutants was uniquely restricted to the cytoplasm, with little to no expression observed in nuclear fractions (Fig. 2A and B). Moreover, total protein abundance of these mutated menin proteins was significantly lower than wild-type menin and the A541T variant. Subsequent immunostaining for FLAG-epitope expression confirmed a significant increase in cytoplasmic localization of the R516fs and E235K-mutated menin proteins compared with wild-type menin and the A541T variant that maintained strong nuclear and perinuclear expression (Fig. 2C and D).

To confirm that these effects arise from posttranslational deficits as opposed to a deficiency in RNA transcription, we evaluated *Men1* mRNA levels following overexpression of wild-type and mutated menin constructs. MEF*^ΔMen1^* cells showed an approximately 20,000-fold induction in *Men1* mRNA expression compared with empty vector-transfected cells, and expression levels did not significantly differ between wild-type menin and the mutated menin proteins (Fig. 2E). In comparison, introduction of the menin constructs in wild-type MEF cells resulted in just an approximately 2-fold increase in *Men1* mRNA expression, showing this cell line to be more resistant to menin overexpression (Fig. 2F). In summary, we concluded that reduced nuclear and cellular expression of the R516fs and E235K mutants resulted from accelerated turnover rather than an impairment in transcription.

### R516fs and E235K Mutations Exhibit Reduced Protein Stability and Shortened Half-lives Compared with Wild-type Menin

**FIGURE 3:**
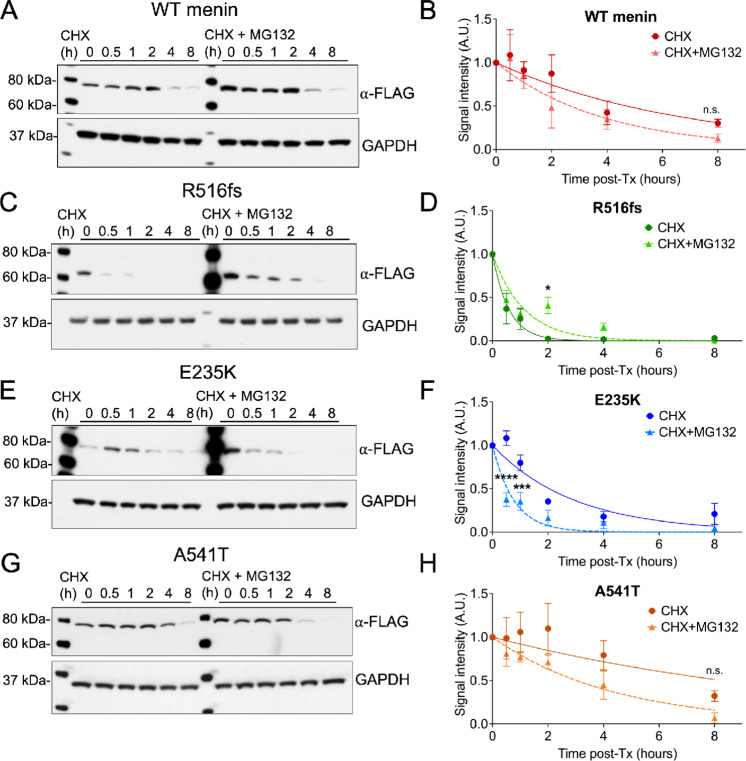
R516fs and E235K mutations exhibit reduced protein stability and shortened half-lives compared with wild-type (WT) menin. **A,** Western blot analysis of MEF*^ΔMen1^* whole-cell extracts following overexpression of wild-type menin in the presence of the protein synthesis inhibitor cycloheximide (CHX, 10 μmol/L) alone or pretreated with the proteosome inhibitor MG132 (10 μmol/L). **B,** Normalized FLAG band signal intensity plotted as a function of time in the presence of CHX with and without MG132 pretreatment. Overexpression of the R516fs (**C**–**D)**, E235K(**E, F**), and A541T (**G, H**) menin proteins with normalized FLAG band signal intensity plotted as a function of time in the presence of CHX alone or in combination with MG132 pretreatment. *n* = 3 experimental replicates, mean ± SEM. *, *P* < 0.05; ***, *P* < 0.01; ****, *P* < 0.0001 by two-way ANOVA.

We next determined whether reduced and cytoplasmic expression of the R516fs and E235K mutants resulted from accelerated turnover of these mutants. The half-life of menin proteins was evaluated in MEF*^ΔMen1^* cells following transient overexpression of wild-type menin or the three mutants in the presence of cycloheximide, to inhibit protein synthesis. Consistent with previous reports (13), reduced cellular expression of wild-type menin was observed by 4 hours (Fig. 3A). Pretreatment with the proteosome inhibitor MG132 resulted in a slight reduction in the half-life of wild-type menin (from 4 to 3 hours), suggesting that turnover of the wild-type protein was insensitive to proteosome blockade (Fig. 3A and B). In striking contrast, expression of the R516fs mutant was rapidly lost within 0.5 hour of cycloheximide and pretreatment with MG132 increased the protein half-life (Fig. 3C and D). Consistent with its reduced cellular expression, the E235K mutant showed a significantly reduced half-life compared with wild-type menin (2 hours). However, unlike the R516fs mutant, proteosome inhibition did not rescue the half-life of the E235K mutant (Fig. 3E and F). Overexpression of the A541T variant in the presence of cycloheximide showed this variant to be more resistant than wild-type menin to protein turnover, with its half-life determined at 6 hours. Moreover, pretreatment with MG132 reduced the half-life of this variant to that of wild-type menin (∼3.5 hours; Fig. 3G and H). In summary, both R516fs and E235K-mutated proteins exhibit a significant reduction in half-life compared with wild-type menin at baseline expression, and there was no significant difference in the half-life of the A541T variant with the wild-type protein. Therefore, the frameshift and exon 4 point mutations were the most unstable of the missense mutations and they surprisingly show opposing responses to proteosome inhibition. Whereas the half-life of the R516fs mutant is rescued by proteosome inhibition, the lack of response by the E235K mutant suggested a different mechanism that mediates protein turnover.

### Differential Expression of Mutated Menin Proteins is Conserved in Gastrin-expressing Tumor Cell Lines

**FIGURE 4:**
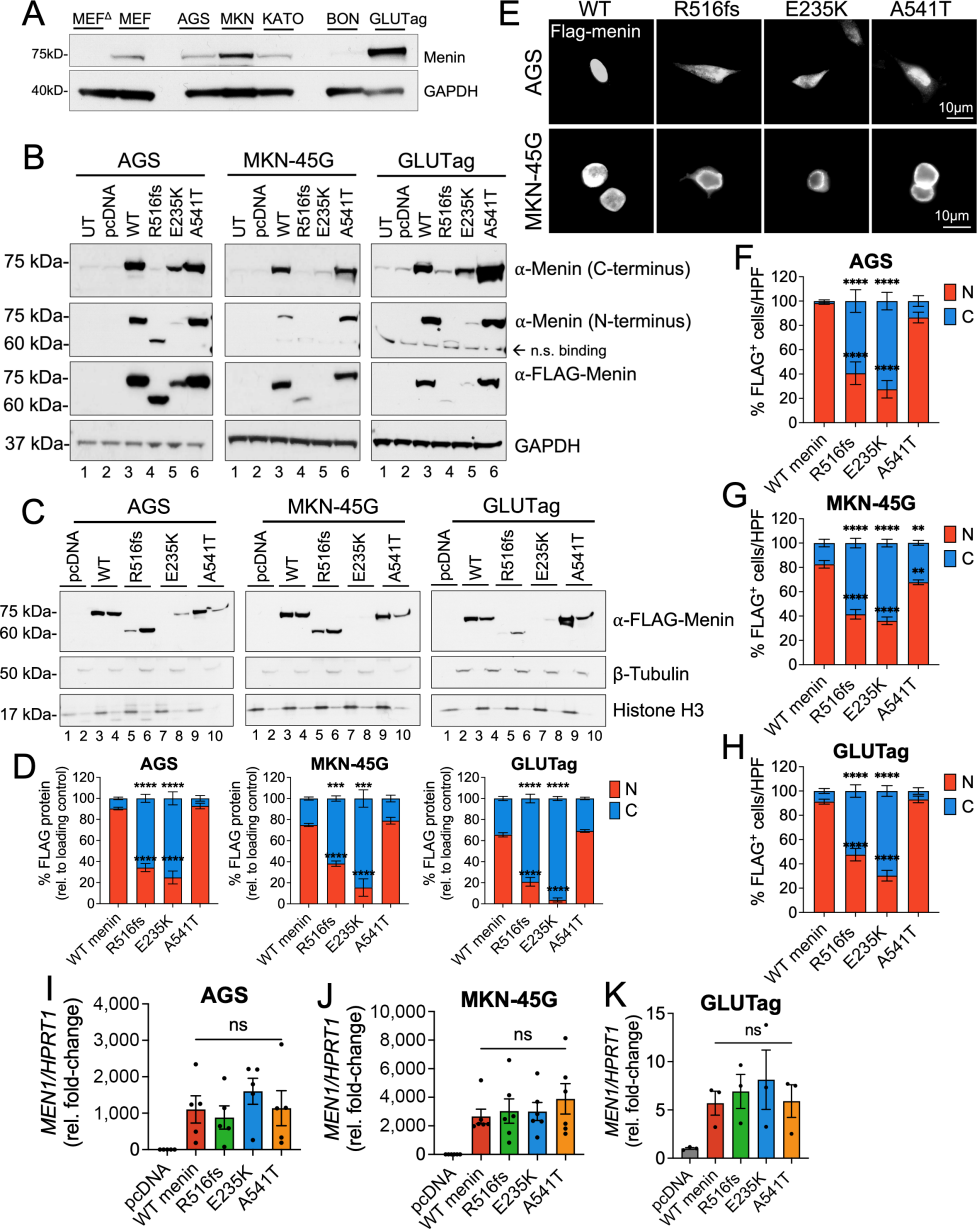
Differential expression of menin mutants is conserved in gastrin-expressing tumor cell lines. **A,** Western blot analysis of endogenous menin protein expression in MEF*^ΔMen1^*, MEF wild-type, AGS, MKN-45G, KATO III, BON-1, and GLUTag cell lines. **B,** Western blot analysis of menin and FLAG expression in whole-cell extracts from AGS, MKN-45G, and GLUTag cells following overexpression of empty vector (pcDNA, lane 2), wild-type menin (lane 3), and the three mutated menin proteins (lanes 4–6). Untransfected cells (UT) are shown in lane 1. **C,** Western blot analysis of FLAG expression in nuclear and cytoplasmic protein extracts in AGS, MKN-45G, and GLUTag cells. Nuclear fractions are shown in lanes 1, 3, 5, 7, and 9 while cytoplasmic fractions are shown in lanes 2, 4, 6, 8, and 10. Histone H3 and β-tubulin were used as loading control markers for nuclear and cytoplasmic fractions, respectively. **D,** Quantitation of FLAG protein band intensity in nuclear and cytoplasmic compartments normalized to respective loading controls. *n* = 3 experimental replicates; ***, *P* < 0.001; ****, *P* < 0.0001 by two-way ANOVA with Tukey *post hoc* test; mean ± SEM. **E,** Immunofluorescent images of FLAG-stained AGS and MKN-45G with FLAG-Menin shown in white. Scale bar = 10 μm. Quantitation of FLAG expression in nuclear and cytoplasmic compartments expressed as a percentage of positively transfected AGS (**F**), MKN-45G (**G**), and GLUTag (**H**) cells. Ten images taken at 200X magnification were counted across *n* = 3 experiments for a total of 30 images per group; *, *P* < 0.05 by two-way ANOVA with Tukey *post hoc* test; mean ± SEM. Relative menin mRNA expression in AGS (**I**), MKN-45G (**J**), and GLUTag (**K**) cells 48 hours following overexpression of menin plasmid constructs. WT, wild-type.

As the previous studies examined the structural effects of *MEN1* mutations and the germline variant in a *Men1*-null murine stromal cell line, we next tested menin turnover in menin competent tumor cell lines. LOH at the *MEN1* locus is reported to occur in less than 50% of duodenal gastrinomas (11), thus, we performed the subsequent structure–function studies in gastric adenocarcinoma and NET cell lines expressing varying levels of endogenous menin (Fig. 4A). The AGS, MKN-45G, and KATO III human gastric adenocarcinoma cell lines were selected on the basis of strong positive gastrin expression, whereas a gastrin-expressing subclone of the GLUTag murine enteroendocrine tumor cell line was used as model of NETs because is secretes hormones including gastrin (33). In addition, we included the BON-1 human PNET cell line in later studies to better evaluate these mutations in the context of NET development.

To further demonstrate that detection of the mutated menin proteins with FLAG and endogenous antibodies coincided, we analyzed their expression using commercially available menin antibodies raised against both C- and N-terminal peptide regions. The C-terminal menin antibody recognizes aa 575–615 (34–36), whereas the N-terminal antibody recognizes aa 1–300 of the human peptide sequence (34, 37). As expected, only the N-, and not C-terminal menin antibody detected the truncated R516fs mutant at a lower molecular weight compared with wild-type menin and the E235K and A541T proteins (Fig. 4B). Both wild-type and the A541T variant showed robust overexpression compared with the R516fs and E235K mutants irrespective of using menin and FLAG antibodies for protein detection (Fig. 4B). Similar to the previous observations in MEF*^ΔMen1^* cells, wild-type menin protein localized to both the nuclear and cytoplasmic compartments in all three tumor cell lines; however, expression was predominantly nuclear (90% in AGS, 80% in MKN-45G, 65% in GLUTag; Fig. 4C and D). A similar subcellular expression pattern was maintained by the A541T variant. In contrast, the R516fs mutant showed significantly reduced expression in nuclear fractions (35% in AGS, 40% in MKN-45G, 20% in GLUTag). Of the three mutated proteins, the E235K mutant retained the lowest nuclear expression (30% in AGS, <20% in MKN-45G, 5% in GLUTag; Supplementary Fig. S1A). Immunofluorescence staining for FLAG epitope expression further confirmed predominantly cytoplasmic expression of the R516fs and E235K-mutated menin proteins in AGS, MKN-45G, and GLUTag tumor cell lines (Fig. 4E–H; Supplementary Fig. S1B). Thus, the R516fs and E235K mutants maintained cytoplasmic-dominant location and reduced cellular expression in tumor cell lines with low endogenous menin expression. We further confirmed that *MEN1* transcript levels did not significantly differ between wild-type menin and the mutated proteins in AGS, MKN-45G, and GLUTag cells (Fig. 4I–K). Despite observing no difference in the expression of menin mRNA levels among transfected cells, we observed a large variation in menin mRNA expression in cell lines expressing endogenous menin protein. This variation correlated with higher endogenous protein expression, with the greatest mRNA variability observed in the GLUTag cells expressing the highest levels of menin protein (Fig. 4A and K). In summary, altered subcellular expression of the R516fs and E235K-mutated proteins was attributed to impairment in protein processing and accelerated turnover of mutated menin proteins rather than a deficiency at the transcriptional level.

### Clinically Defined *MEN1* Mutations Exhibit Loss of Growth-suppressive Function in Tumor Cell Lines

**FIGURE 5:**
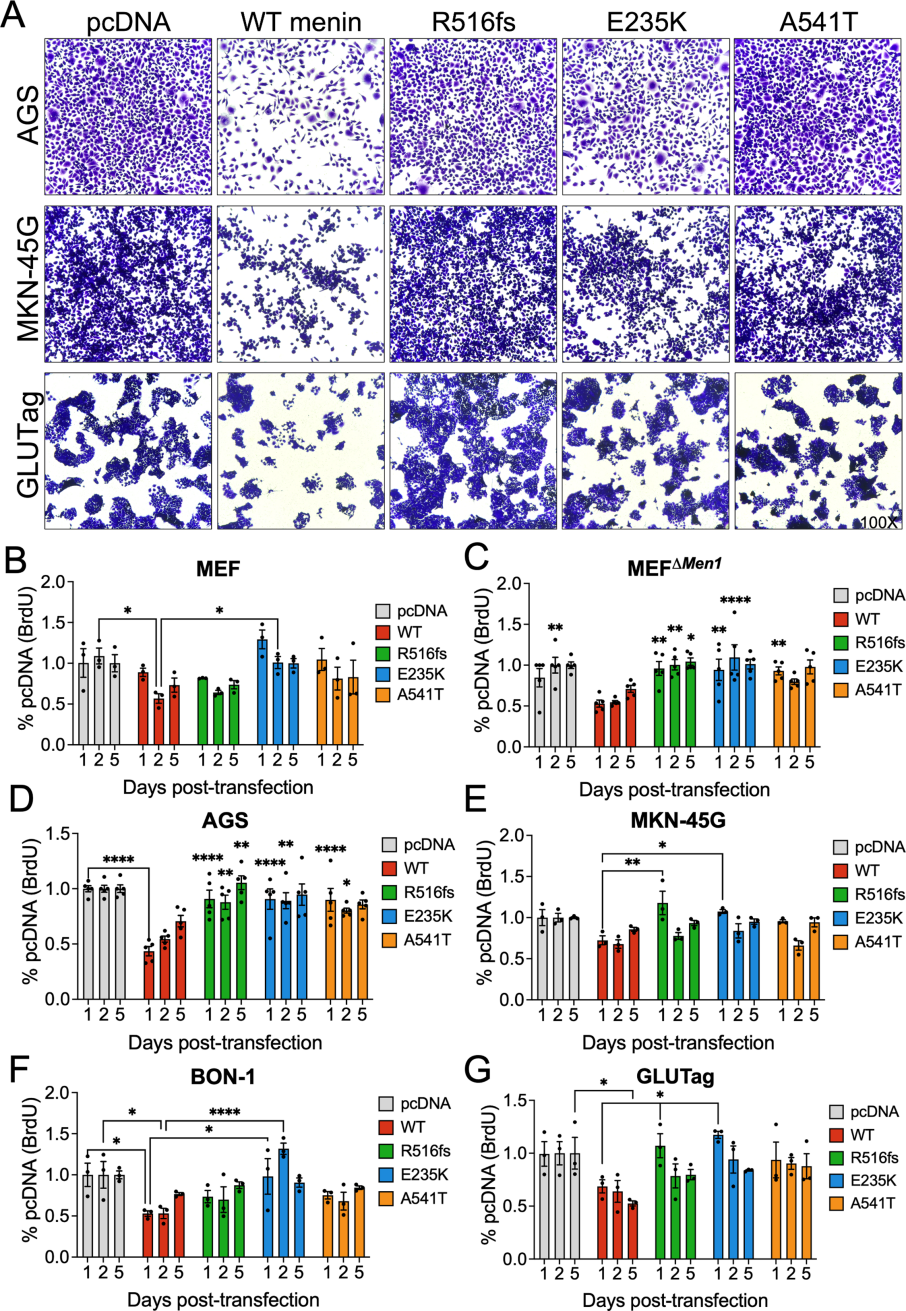
Clinical *MEN1* mutations and variants exhibit loss of growth-suppressive function in a cell-dependent manner. **A,** Representative bright-field images of crystal violet-stained AGS, MKN-45G, and GLUTag following 72 hours transfection with empty vector (pcDNA), wild-type (WT) menin, and the three mutated menin proteins. Images taken at 100X magnification. BrdU incorporation was assayed 1, 2, and 5 days posttransfection in MEF (**B**), MEF*^ΔMen1^* (**C**), AGS (**D**), MKN-45G (**E**), BON-1 (**F**), and GLUTag (**G**) cell lines. *n* = 3–5 experimental replicates. Asterisks represent comparisons between each timepoint. *, *P* < 0.05; **, *P* < 0.01; ****, *P* < 0.0001 by two-way ANOVA with Tukey *post hoc* test; mean ± SEM.

Following confirmation that these structural deficits were recapitulated in multiple hormone-expressing tumor cell lines, we investigated whether these clinically defined *MEN1* mutations and variant confer pathogenic properties by inhibiting the tumor-suppressive function of wild-type menin. Using *in vitro* cell growth assays, we assessed whether the three mutated menin proteins retain the ability to suppress cell proliferation. As anticipated, overexpression of wild-type menin significantly inhibited the proliferation of multiple tumor cell lines as visualized by crystal violet staining (Fig. 5A). Compared with the menin-competent MEFs, MEF*^ΔMen1^* cells showed a more robust response to growth inhibition by wild-type menin, as anticipated (Fig. 5B and C). Whereas wild-type menin was shown to reduce BrdU incorporation by menin-null cells, the three mutated menin proteins showed significant loss of function in suppressing BrdU uptake (Fig. 5C). Similarly, all three menin mutants showed a strong reduction in their ability to suppress proliferation in the AGS cell line, with the R516fs mutation exhibiting the most significant and sustained loss of function (Fig. 5D). In comparison, the R516fs and E235K mutants showed significant loss of function in the MKN-45G cell line, while the A541T variant showed no significant effect on BrdU uptake (Fig. 5E). Interestingly, the somatic E235K mutant originally identified in a PNET showed the greatest loss of growth-suppressive function in the BON-1 NET cell line (Fig. 5F); however, the effect of the germline and DGAST-specific R516fs mutation showed no appreciable effect. In contrast, both the R516fs and E235K mutants showed significant loss of growth-suppressive function in the GLUTag tumor cell line (Fig. 5G). Taken together, these observations suggest a pathologic role for these clinically defined *MEN1* mutations and variant irrespective of apparent structural deficits leading to altered subcellular expression. Moreover, these results demonstrate the potential for context-specific regulation as the apparent loss of function demonstrated by the three mutated menin proteins was dependent on the cell type.

### Clinically Defined *MEN1* Mutations and the Germline Variant Reduce the Ability of Menin to Repress Gastrin Gene Expression

**FIGURE 6:**
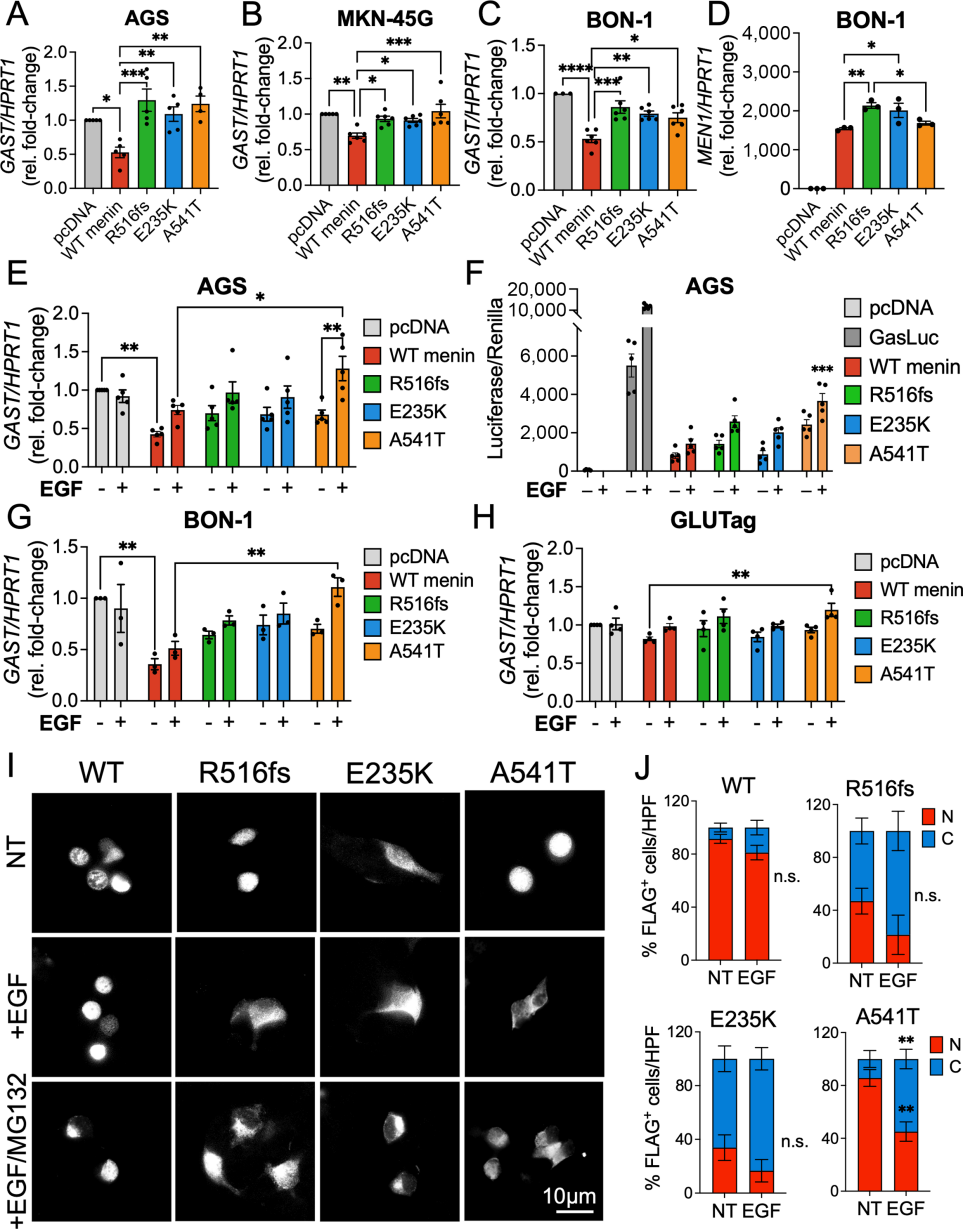
Clinical *MEN1* mutations and variants reduce the ability of menin to repress gastrin gene expression. Gastrin transcript levels (*GAST*) in AGS (**A**), MKN-45G (**B**), and BON-1 (**C**) cells following overexpression with empty vector (pcDNA), wild-type menin, and the three menin mutants. *GAST* mRNA was normalized to *HPRT1* expression and reported as fold-change relative to pcDNA control. **D,** Expression of MEN1 mRNA in BON-1 cells. *, *P* < 0.05; **, *P* < 0.01; ***, *P* < 0.001; ****, *P* < 0.0001 by two-way ANOVA with Tukey *post hoc* test; mean ± SEM. **E,** Relative GAST mRNA expression in AGS cells overexpressing menin plasmids and following serum starvation and treatment with EGF (16 hours, 40 nmol/L). **F,** The GasLuc system was used to evaluate human gastrin promoter activity following overexpression of empty vector (pcDNA), GasLuc plasmid alone, or GasLuc plasmid in the presence of wild-type menin and the three mutants and EGF (16 hours, 40 nmol/L). Firefly luciferase activity was normalized to the *Renilla*-Luciferase co-reporter. ***, *P* < 0.0001, by one-way ANOVA with Tukey *post hoc* test; mean ± SEM. Relative *GAST* mRNA expression in BON-1 (**G**) and GLUTag (**H**) cells overexpressing menin plasmids and following serum starvation and treatment with EGF (16 hours, 40 nmol/L). **I,** Immunofluorescent staining of FLAG-menin expression in GLUTag cells in the presence or absence of EGF (8 hours, 40 nmol/L) and MG132 (10 μmol/L). NT = no treatment control. **J,** Quantitation of FLAG-menin expression in nuclear and cytoplasmic compartments from immunofluorescence-stained images. Counts were obtained from 10 random HPF images. **, *P* < 0.01 = by two-way ANOVA with Tukey *post hoc* test; mean ± SEM. WT, wild-type.

Consistent with its ability to repress gastrin gene expression (14), a heterozygous *MEN1* variant and two heterozygous mutations were identified in all patients diagnosed with duodenal and pancreatic gastrinomas in our previously published cohort (20). Thus, we determined whether these clinical mutations and variant conferred loss of function in repressing gastrin expression during the pathogenesis of these tumors. Gastrin transcript levels were evaluated in gastrin-expressing AGS, MKN-45G, and BON-1 tumor cell lines following transient overexpression of wild-type and menin mutant proteins. As anticipated, wild-type menin significantly repressed gastrin mRNA expression at 72 hours (Fig. 6A–C). In comparison, the two menin mutations and variant showed significant functional loss in repressing gastrin mRNA expression. As the BON-1 cells showed the lowest expression of endogenous menin protein among the gastrin-expressing cell lines, we examined whether BON-1 cells exhibited similar variability in *MEN1* mRNA expression following plasmid overexpression. Surprisingly, we observed significantly less variation in *MEN1* mRNA expression in subsequent experimental transfections in the BON-1 cell line, suggesting that the presence of endogenous menin protein contributes to variability in the expression of wild-type and mutated menin proteins (Fig. 6D).

Given that the A541T variant showed a surprising loss of function in the absence of overt changes in subcellular expression, we posited that additional regulation by extracellular growth factors might contribute to the loss of function shown by this variant. Specifically, the replacement of an alanine to a threonine at residue 541 introduces a potential phosphorylation site proximal to the NLS sequence in Exon 10, and we hypothesized that this variant may render menin more sensitive to posttranslational regulation by signaling kinases. To test this further, AGS cells overexpressing the respective menin constructs were serum starved and treated with human recombinant EGF (40 nmol/L) for 16 hours and subsequently evaluated for gastrin mRNA expression. EGF concentration was based on previously reported ranges known to induce gastrin gene expression in cultured endocrine cells (26). We observed a striking loss of function by the A541T variant upon induction with EGF (Fig. 6E). We next evaluated gastrin promoter activity using a previously characterized GasLuc plasmid carrying 240 bp of the human gastrin gene (25) cloned upstream of the firefly luciferase (Luc) gene (14). AGS cells were transiently transfected with menin expression vectors in the presence of the GasLuc reporter plasmid. The transfected R516fs mutant and A541T variant showed partial loss of function in suppressing GasLuc activity, whereas the E235K mutant behaved similarly to wild-type menin. In addition, EGF treatment significantly induced the expression of the 240 bp human gastrin promoter and Luc reporter activity in the presence of the A541T variant compared with the wild-type protein (Fig. 6F). Because GasLuc activity and gastrin mRNA levels showed some degree of variation in the presence of the mutated proteins, it remains plausible that menin differentially binds to the 240 bp gastrin promoter compared with the endogenous full-length promoter. To test the robustness of our observations, we assessed gastrin mRNA expression in BON-1 and GLUTag cells following treatment with EGF. As anticipated, the A541T variant showed the greatest loss of gastrin-suppressive function compared with wild-type menin and the R516fs and E235K mutants (Fig. 6G and H). Intriguingly, the GLUTag cell line showed no significant response in gastrin regulation by wild-type menin or the mutated proteins, likely as a result of the high endogenous menin expression confirmed previously. Finally, we sought to investigate whether the loss of suppressive function by the A541T variant results from EGF-induced nuclear export. FLAG-menin expression was visualized in GLUTag cells overexpressing the respective menin plasmids in the presence or absence of EGF and the proteosome inhibitor MG132 (Fig. 6I; Supplementary Fig. S1C). Quantitative analysis showed a significant loss of nuclear FLAG-menin expression in the A541T variant upon EGF induction (Fig. 6J). Thus, we conclude that the previous loss of function observed in the A541T variant may be explained by additional regulatory factors, including activation of growth factor signaling pathways that converge on the NLS sequence and regulate the function of the A541T variant.

### Treatment with the Menin-MLL Inhibitor MI-503 Rescues Nuclear Menin Expression and Function

**FIGURE 7:**
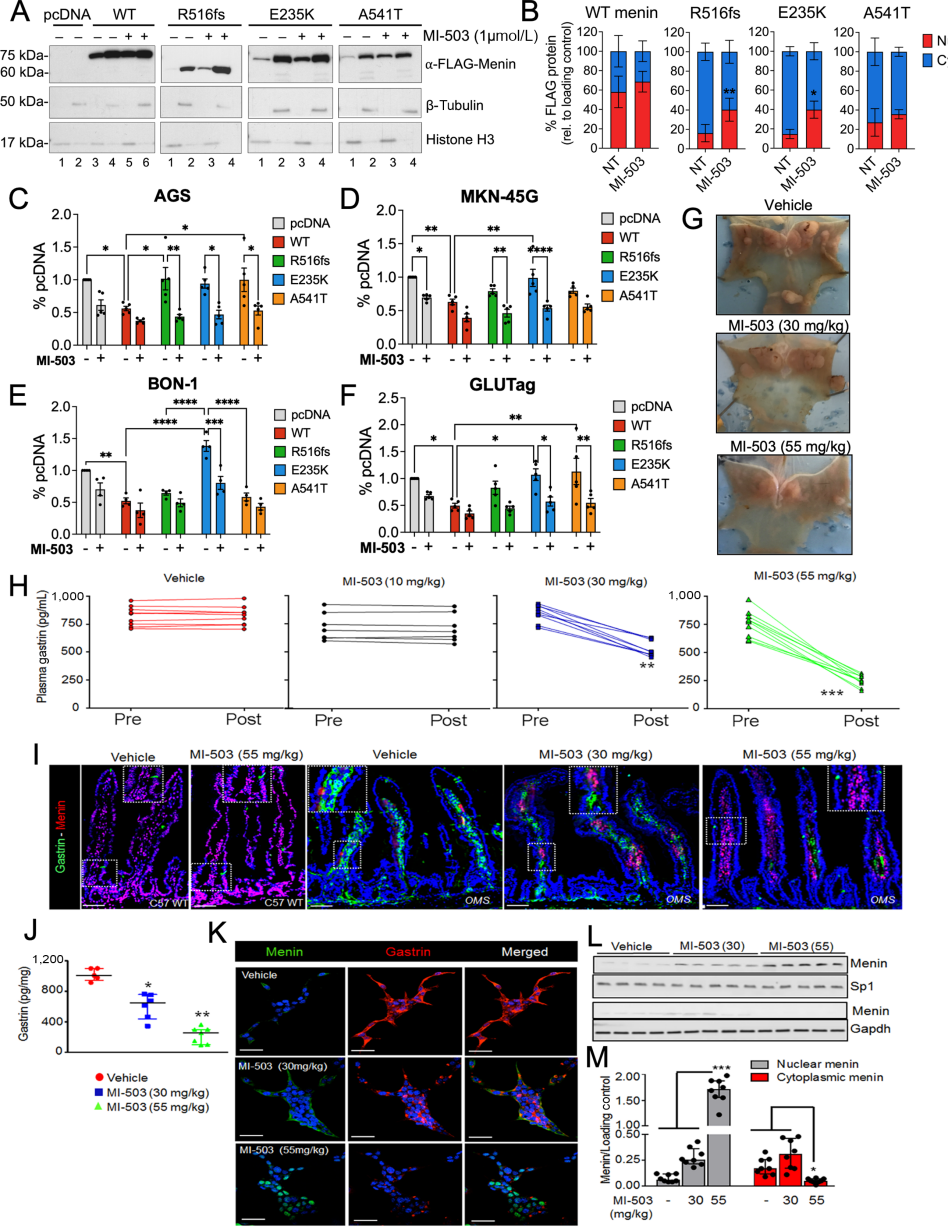
Treatment with the menin-MLL inhibitor MI-503 rescues nuclear menin expression and function. **A,** Western blot analysis of nuclear and cytoplasmic extracts of transfected AGS cells treated with vehicle or MI-503 (48 hours, 1 μmol/L). Histone H3 and β-tubulin were used as loading control markers for nuclear and cytoplasmic fractions, respectively. **B,** Quantitation of FLAG protein band intensity in nuclear and cytoplasmic compartments normalized to respective loading controls. *n* = 4 experimental replicates; *, *P* < 0.05 by two-way ANOVA with Tukey *post hoc* test; mean ± SEM. BrdU incorporation in transfected AGS (**C**), MKN-45G (**D**), BON-1 (**E**), and GLUTag (**F**) cells treated with vehicle (DMSO) or MI-503 (48 hours, 1 μmol/L). *, *P* < 0.05 by two-way ANOVA with Tukey *post hoc* test; mean ± SEM. **G,** Representative macroscopic images of *OMS* mice stomachs following treatment with vehicle or 30 and 55 mg/kg of MI-503. Arrows indicate to gastric NETs. **H,** Enzyme immunoassay measurement of plasma gastrin concentration from *OMS* mice before (Pre) and after (Post) treatment with vehicle, 10, 30, or 55 mg/kg of MI-503 for 4 weeks. *n* = 10–12 mice; **, *P* < 0.01 by paired *t* test; mean ± SEM. **I,** Immunofluorescence-stained images of gastrin (green) and menin (red) expression in the proximal duodenum of C57 wild-type mice versus OMS mice treated with vehicle or MI-503 at the indicated doses for 4 weeks. Boxes indicate restoration of nuclear menin expression and reduced gastrin expression restricted to the lamina propria. **J,** Enzyme Immunoassay (EIA) measurement of intracellular gastrin content of glial cultures isolated from omeprazole treated *Men1^ΔIEC^;Sst^−^^/^^−^* mice after treatment with vehicle, 30, or 55 mg/kg MI-503 for 4 weeks (*n =* 6–8 mice), expressed after normalization to total cellular protein content. **K,** Immunofluorescent staining of gastrin (red) and menin (green) in glial cultures isolated from duodenal lamina propria of OMS mice and then treated *ex vivo* with vehicle, 0.1, or 5 nmol/L MI-503 for 2 days. **L,** Representative western blot showing Menin expression in total cell lysates from duodenal glial cultures (from *OMS* mice) treated with MI-503 for 2 days. *n* = 4–5 mice. **M,** Quantitation of menin protein levels on blots as a function of MI-503 dose. *n* = 8 mice. WT, wild-type.

As the previously observed loss of function in the R516fs and E235K mutants corresponds with impaired nuclear localization of menin, we posited whether stabilizing the expression of these mutated proteins might rescue menin function. Menin-MLL (KMT2A) inhibitors (MI), such as MI-503, are small-molecule compounds that have been shown to bind directly to menin and induce thermal stabilization (38). Therefore, we tested the effect of MI-503 on menin expression in AGS cells overexpressing wild-type menin and the three mutated proteins. Compared with vehicle treatment, MI-503 increased the expression of nuclear menin, with the greatest effect observed in R516fs and E235K mutants (∼2-fold increase; Fig. 7A and B). We further evaluated whether increased menin expression rescued its ability to suppress tumor cell proliferation. Indeed, treatment of AGS, MKN-45G, BON-1, and GLUTag cells with MI-503 significantly reduced BrdU uptake in the presence of the R516fs and E235K mutations and A541T variant (Fig. 7C–F).

Finally, we tested whether MI-503 blocks NET development and suppresses hypergastrinemia *in vivo* by using a previously reported transgenic mouse model of gastric NET development (12). Here, the *Cre* recombinase enzyme was expressed downstream of the *Villin* promoter specifically marking intestinal epithelial cells. The *Villin Cre; Men1^FL/FL^* mice show biallelic deletion of the *Men1* gene in intestinal epithelium as reported previously. *Villin Cre; Men1^FL/FL^* mice fed an omeprazole-laced chow (OMS mice) develop hypergastrinemia, enterochromaffin-like cell hyperplasia, and gastric NETs by 6–12 months of age (12). As reported previously, hypergastrinemia and gastric NETs develop secondary to the emergence of hyperplastic gastrin-expressing enteric glial cells in the duodenal lamina propria. Three doses (10, 30, 55 mg/kg) of MI-503 were administered to OMS mice by daily intraperitoneal injections for 4 weeks. By 12 months of age, mice showed reduced gastric hyperplasia in the corpus (Fig. 7G). Consistent with this, mice exhibited a dose-dependent decrease in plasma gastrin with a maximum 70% decrease observed with the 55 mg/kg dose (Fig. 7H). Menin protein increased in the lamina propria and coincided with reduced gastrin staining (Fig. 7I). Isolating enteric glial cells from the MI-503-treated *OMS* mice permitted determination of the gastrin content and demonstrated a significant 70% reduction in the circulating peptide at the 55 mg/kg dose (Fig. 7J and K). A dose-dependent reexpression of menin was also observed in the glial cultures and coincided with reduced gastrin expression (Fig. 7L). Menin was retained in the nuclear fractions and was barely detectable in the cytoplasmic fractions of glial cells isolated from MI-503–treated *OMS* mice, compared with that of vehicle-treated controls (Fig. 7L and M). Thus, MI-503 rescued the nuclear expression and function of mutated menin proteins in multiple hormone-expressing tumor cell lines and blocked hypergastrinemia-induced gastric NET development in mice.

## Discussion

Following its original discovery as a tumor suppressor gene (21, 39), over 1,700 mutations have been mapped to the coding region of the *MEN1* locus (8). Despite a clear association between the occurrence of inactivating *MEN1* mutations and tumor onset in cases of sporadic and hereditary MEN1 syndrome, knowledge of how these mutations manifest during pathogenesis remains limited. *MEN1* mutations are dispersed throughout its nine coding exons and fail to cluster into specific functional domains of its protein product menin, making it difficult to discern a causal relationship with the location of any given mutation. A prior study of 169 sporadic PNETs identified alterations in menin protein expression in the vast majority of cases (80%), with nuclear menin expression absent in tumors harboring truncating *MEN1* mutations, and strong cytoplasmic reactivity in PNETs carrying *MEN1* missense mutations (28). Given that LOH at *MEN1* loci occurs in less than 50% of MEN1-gastrinomas (11), we investigated the possibility that alternative posttranslational mechanisms regulate menin protein expression in the context of incomplete LOH. We sought to determine whether clinically defined heterozygous *MEN1* mutations predispose menin to posttranslational regulation that leads to loss of nuclear expression and subsequent functional inactivation.

Here, we studied the structural and functional implications of two clinical *MEN1* mutations and a third germline variant that we identified in a cohort of 10 patients with confirmed GEP-NETs (20). Our rationale for selecting these specific mutations and variant centered on their proximity to NLS, in addition to the presence of amino acid substitutions that confer significant structural changes or introduce the potential for posttranslational modification. Of these, we examined a germline c.1546dupC frameshift insertion that leads to the expression of a premature stop codon (R516fs) before the final two NLS sequences and encodes a truncated menin protein. The c.703G>A somatic missense mutation leads to a glutamic acid to lysine exchange (E235K) and we reasoned that a substitution in polar groups might render a significant structural change near the “central binding pocket.” Finally, the c.1621G>A germline variant that was identified across our cohort has also been reported on extensively, with conflicting studies suggesting both a benign or a pathogenic role in predisposing patients toward NET development (28–30, 40). The mutations and variant in the patient tumors were identified as heterozygous. The A541T variant was first reported as a benign polymorphism occurring with a frequency of 4% (GCA > ACA) in American-based studies and approximately 1%–2% of the general European population are carriers of the resulting A541T SNP. As poignantly highlighted by Bazzi and colleagues, more recent analyses in a French population identified this variant only in affected individuals with clinical MEN1 features and indicated a lower prevalence in the general population than suggested previously (28, 40, 41). It is important to recognize that the incidence of the heterozygous p.A541T and p.T541A variants vary according to the population being examined, and thus it is critical to distinguish which of the two *MEN1* sequences predominate in a given study population. Given that the current study focuses on the A541T variant previously identified in GEP-NET–bearing patients, we posited that the exchange of an alanine to threonine residue immediately upstream to the NLSa sequence creates a potential phosphorylation site for posttranslational modification. As prior studies have shown that menin is regulated by phosphorylation in response to external stimuli, such as DNA damage (31), we considered whether the A541T missense variant might confer a similar regulatory mechanism leading to loss of nuclear menin expression and functional inactivation.

In our study, the R516fs and E235K mutants exhibited severe defects in the expression and half-life of menin protein. Impaired expression coincided with a significant loss of function in suppressing cell proliferation and gastrin gene expression, with the greatest effect observed with the R516fs and E235K-mutated proteins. Surprisingly, we found that the germline A541T variant conferred loss of function that was dependent on the cell type despite sharing a similar expression pattern and half-life to wild-type menin protein. As the A541T variant introduces a potential phosphorylation site at threonine 541, and this single-nucleotide variant occurs proximal to the NLS sequence in Exon 10, we posited that this variant might render menin more sensitive to regulation by signaling kinases. Indeed, we observed that activation by EGF stimulates loss of gastrin-suppressive function by the A541T variant, and this coincided with increased nuclear-to-cytoplasmic shuttling of menin. Thus, the presence of activated kinase signaling networks in different tissues might also explain the cell type–dependent variation in loss of function exhibited by the A541T variant.

Collectively, our findings are consistent with previous studies indicating that *MEN1* missense mutations are more susceptible to degradation compared with wild-type menin protein (41–43). Moreover, we show that altered nuclear expression and loss of function exhibited by these mutants and variant are conserved across stromal cells and hormone-expressing cell lines with epithelial and enteroendocrine characteristics. The three mutated menin proteins exerted differential effects depending on the cell line in which they were overexpressed and our observations point to a role for endogenous menin in modulating these variations. This raises the potential for context- and tissue-specific regulation that might inform the penetrance of inactivating mutations at different tissue sites. Indeed, our observations suggest that the E235K and A541T-mutated menin proteins may undergo degradation via a noncanonical pathway independent of ubiquitin-mediated degradation by the proteosome.

Prior studies focused on mapping structural defects at the protein level have shown that mutations arising in specific functional domains of menin can impair protein–protein interactions that may result in altered gene expression leading to cell proliferation, altered cell specification, and NET development (25, 42–44). For instance, a recent *in silico* analysis of 253 disease related *MEN1* missense mutations identified three single-nucleotide mutations that impaired the binding affinity of menin with the mixed lineage leukemia 1 (MLL) protein (45). In contrast, these mutations did not have an apparent effect on the menin–JUND interaction, further raising the potential for context-specific regulation that might dictate the tumor-suppressive and oncogenic properties of menin. Given that menin is known to recruit JUND to the gastrin promoter and suppress gastrin gene expression (14), future co-immunoprecipitation studies are required to determine whether these mutations preclude menin from interacting with known binding partners, including JUND and MLL1. This knowledge will be critical to informing whether rescuing mutated menin expression restores its interaction with protein binding partners. Finally, it should be noted that these studies are limited to standalone observations of a single given mutation. As modeled by Knudsons’ “two hit” hypotheses, examining whether germline and acquired mutations exert an additive or synergistic effect is critical to develop a comprehensive understanding of neoplastic transformation and pathogenesis.

The significance of this work is emphasized by the fact that no targeted therapeutic currently exists for patients with MEN1 syndrome. MEN1-associated neoplasms generally display a more aggressive clinical phenotype (e.g., multifocal tumors), yet surveillance in this population remains insufficient as upward of 60% of MEN1-gastrinoma cases present with metastases upon diagnosis (7). Small molecules that specifically prevent the nuclear export of menin and its subsequent degradation in the cytoplasm, in contrast to simply inhibiting the proteasome might be a promising therapeutic approach to increase nuclear menin levels and mediate suppression of gastrin expression. Menin-MIs are small molecules that bind directly to menin at residues F9 and P13 (45, 46) where both JUND and MLL bind. While MIs have been most extensively studied in leukemias where menin functions as a co-oncoprotein (47), MIs have also shown efficacy in blocking proliferation of solid tumors, including prostate and hepatocellular carcinoma (36, 48). To our knowledge, the delivery of MIs in human and animal NET models has not yet been investigated, thus emphasizing the potential translational relevance of the current study. Consistent with the latter research showing MI treatment increases menin protein levels (47), we observed a similar effect in a gastrin-expressing AGS cells overexpressing mutated menin proteins. The AGS cells used to study the mutated menin proteins express low endogenous menin at baseline. Transfection of the mutated menin proteins in AGS cells shows that these three mutated menin proteins, including the most unstable of these, are expressed at astonishingly higher levels compared with the endogenous protein. Thus, we attribute the increase in menin expression by MI-503 to restoration of the mutated protein and not endogenous menin per say. In comparison, endogenous menin expressed by enteric glial cultures from the OMS mice was previously shown to be lost through a non-cell autonomous mechanism and not by biallelic deletion because detection of the protein was reversible. In this scenario, it is likely that the observed increase in menin expression is due to the restoration of endogenous nuclear menin protein following MI-503 treatment.

Thus, our studies support future investigation into MIs as a novel therapeutic approach for targeting GEP-NETs displaying aberrant menin protein expression in the absence of complete LOH. As destabilizing menin mutations are reported across multiple NET types (28–30) and in other malignancies, including gastric cancer (49), a large-scale clinical investigation is warranted to determine whether menin protein stability may be associated with clinical phenotype or aggressiveness of disease.

## Supplementary Material

Supplementary Figure S1 Supplementary Figure 1
