# Supplementary material for: Clinically Defined Mutations in MEN1 Alter Its Tumor-suppressive Function Through Increased Menin Turnover
Source: Cancer Res Commun. 2023 Jul 24;3(7):1318–34. doi: 10.1158/2767-9764.CRC-22-0522 (PMC10364643; doi:10.1158/2767-9764.CRC-22-0522)
Supplement: Supplementary Figure S1 — Supplementary Figure 1 [file crc-22-0522-s01.docx]

**Clinically defined mutations in *MEN1* alter its tumor-suppressive function through increased menin turnover**

**Supplementary Figures**


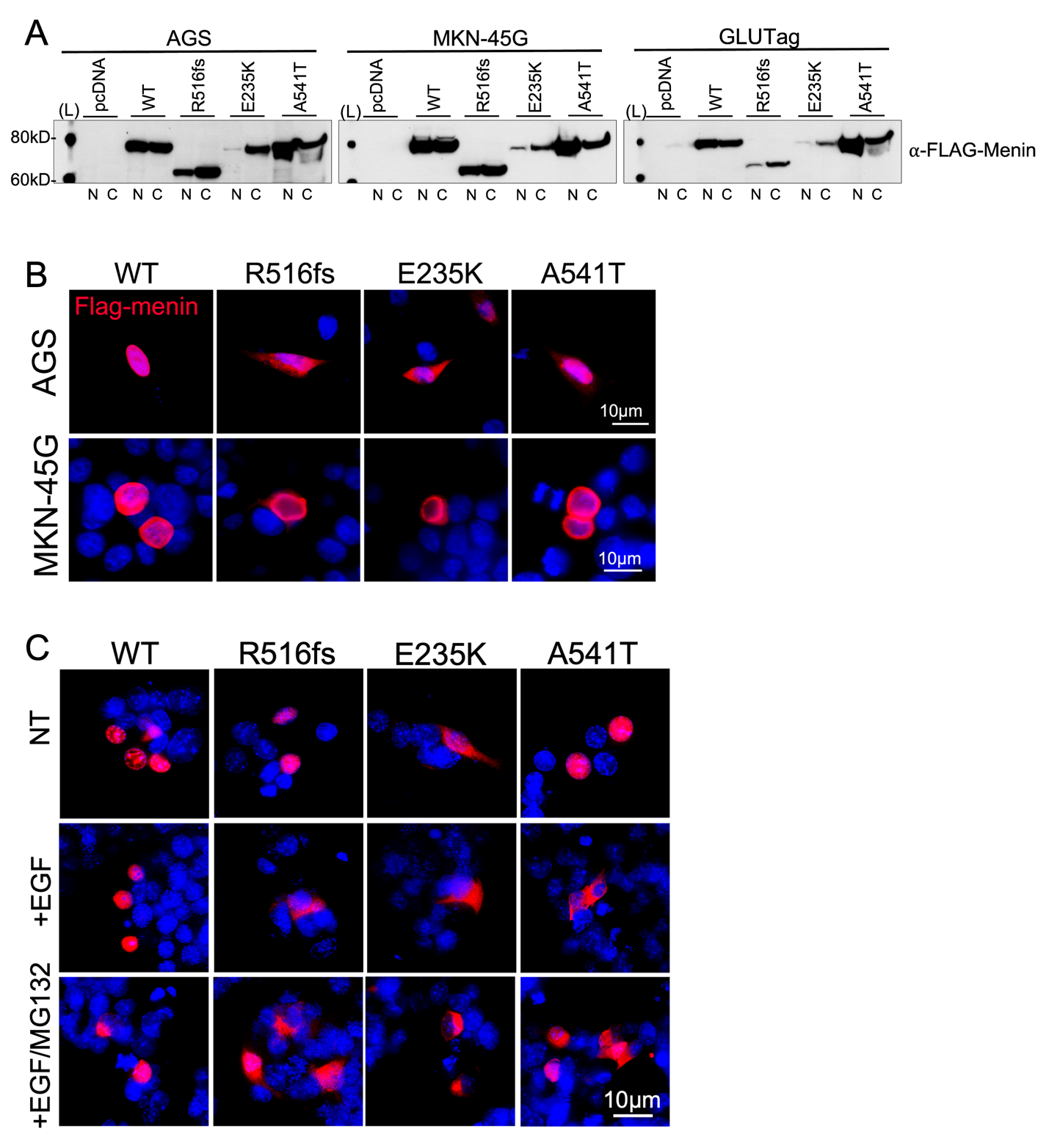


**Supplementary Figure 1.** *(A)* Higher exposure image of the western blot analysis shown in Figure 4C demonstrating faint nuclear menin-FLAG expression in the E235K mutant. Menin-FLAG expression was detected in nuclear (N) and cytoplasmic (C) protein extracts in AGS, MKN-45G, and GLUTag cells. *(B)* Immunofluorescent images of FLAG-stained AGS and MKN-45G cells from Figure 4E showing the overlay with DAPI. FLAG-Menin is shown in red and DAPI in blue. Scale bar = 10 µm. *(C)* Immunofluorescent overlay images of Figure 6I showing FLAG-menin expression (red) and DAPI (blue) in GLUTag cells treated with EGF (8h, 40 nM) and MG132 (10 µM) . NT = no treatment control.
